# Prediction of obesity levels based on physical activity and eating habits with a machine learning model integrated with explainable artificial intelligence

**DOI:** 10.3389/fphys.2025.1549306

**Published:** 2025-07-16

**Authors:** Yasin Görmez, Fatma Hilal Yagin, Burak Yagin, Yalin Aygun, Hulusi Boke, Georgian Badicu, Matheus Santos De Sousa Fernandes, Abedalrhman Alkhateeb, Mahmood Basil A. Al-Rawi, Mohammadreza Aghaei

**Affiliations:** ^1^ Department of Management Information Systems, Faculty of Economics and Administrative Sciences, Sivas Cumhuriyet University, Sivas, Türkiye; ^2^ Department of Biostatistics, Faculty of Medicine, Malatya Turgut Ozal University, Malatya, Türkiye; ^3^ Department of Biostatistics and Medical Informatics, Faculty of Medicine, Inonu University, Malatya, Türkiye; ^4^ Department of Sport Management, Faculty of Sport Sciences, Inonu University, Malatya, Türkiye; ^5^ Yasar Oncan Secondary School, Ministry of National Education, Malatya, Türkiye; ^6^ Department of Physical Education and Special Motricity, Faculty of Physical Education and Mountain Sports, Transilvania University of Braşov, Braşov, Romania; ^7^ Keizo Asami Institute, Federal University of Pernambuco (UFPE), Recife, Brazil; ^8^ Department of Computer Science, Lakehead University, Thunder Bay, Canada; ^9^ Department of Optometry, College of Applied Medical Sciences, King Saud University, Riyadh, Saudi Arabia; ^10^ Department of Ocean Operations and Civil Engineering, Norwegian University of Science and Technology (NTNU), Alesund, Norway; ^11^ Department of Sustainable Systems Engineering (INATECH), Albert Ludwigs University of Freiburg, Freiburg, Germany

**Keywords:** obesity prediction, machine learning, explainable artificial intelligence, physical activity and diet, feature importance

## Abstract

**Objectives:**

This study aims to build a machine learning (ML) prediction model integrated with explainable artificial intelligence (XAI) to categorize obesity levels from physical activity and dietary patterns. The inclusion of XAI methodologies facilitates a comprehensive understanding of the risk factors influencing the model predictions and thus increases transparency in the identification of obesity risk factors.

**Methods:**

Six ML models were used: Bernoulli Naive Bayes, CatBoost, Decision Tree, Extra Trees Classifier, Histogram-based Gradient Boosting and Support Vector Machine. For each model, hyperparameters were tuned by random search methodology and model effectiveness was evaluated by repeated holdout testing. SHAP (SHapley Additive Annotations) and LIME (Local Interpretable Model Independent Annotations) interpretability methods were used to generate local and global feature importance measures.

**Results:**

The CatBoost model exhibited the highest overall performance and achieved superior results in accuracy, precision, F1 score and AUC metrics. Nonetheless, other models such as Decision Tree and Histogram-based Gradient Boosting also yielded strong and competitive results. The results also highlighted age, weight, height and specific food patterns as key predictors of obesity. In terms of interpretability, LIME showed superior in fidelity, whereas SHAP showed improved sparsity and consistency across models, facilitating a comprehensive understanding of trait importance.

**Conclusion:**

This research demonstrates that ML algorithms, when integrated with XAI technologies, can accurately predict obesity levels and explain important contributing risk factors. The use of SHAP and LIME increases model transparency, facilitating the identification of specific lifestyle patterns linked to obesity risk. These findings help to formulate more precise intervention techniques guided by a reliable and understandable predictive framework.

## 1 Introduction

Obesity has become a major global health issue, with its incidence rising rapidly in both developed and developing countries (Mohajan and Mohajan, 2023). The World Health Organization reports that global obesity rates have nearly tripled since 1975, rendering it a significant public health issue of the 21st century (Lim et al., 2020). The syndrome is linked to several comorbidities, such as cardiovascular diseases, type 2 diabetes, specific malignancies, and musculoskeletal ailments, resulting in significant healthcare expenses and diminished quality of life (Adebibe and Coppack, 2022; Afolabi et al., 2023).

Conventional methods for predicting obesity and assessing risk have predominantly depended on rudimentary measurements like Body Mass Index (BMI) and fundamental demographic variables (Liu et al., 2024; Kovacic et al., 2012). These strategies often do not examine the complex interactions among lifestyle factors, physical activity patterns, and dietary habits that lead to the onset of obesity. Additionally, traditional methods may fail to provide specific insights into specific risk variables and possible intervention techniques.

The emergence of machine learning (ML) methods has created new opportunities for more advanced and precise predictive models in healthcare. ML algorithms can analyze extensive amounts of multidimensional data and discern intricate patterns that may not be evident with traditional statistical techniques. Nonetheless, a notable constraint of numerous ML models is their “black box” characteristic, rendering the decision-making process inscrutable to both healthcare personnel and patients (Rudin, 2019; Castelvecchi, 2016).

Explainable Artificial Intelligence (XAI) has arisen as an essential remedy to mitigate this constraint. XAI techniques seek to enhance the transparency and interpretability of ML models, enabling stakeholders to comprehend the rationale behind certain predictions. The transparency offered by XAI is critical in healthcare applications where understanding the logic behind predictions is critical to clinical decision making and patient safety (Angelov et al., 2021; Minh et al., 2022; Khosravi et al., 2022). Despite the potential advantages of combining ML models with XAI approaches in obesity prediction, research adopting this integrated methodology is yet to be widely used. Although numerous studies have investigated ML applications in obesity prediction (Ferdowsy et al., 2021; Dugan et al., 2015; Tanvir et al., 2025; Rodríguez et al., 2021; Singh and Tawfik, 2020; Jeon et al., 2023; Cheng et al., 2021), only a limited number have integrated XAI approaches to yield interpretable outcomes that can inform clinical practice and patient education.

In a similar study predicting obesity levels, the authors achieved 86.5% accuracy using the Random Forest algorithm regardless of BMI parameters (Khater et al., 2024). Our optimized model developed in the current study performed better with an accuracy of 93.67%. M Al-Hazzaa et al., assessed the correlations between obesity markers and various lifestyle factors, including physical activity, sedentary behaviors, and dietary habits, among Saudi adolescents aged 14–19 years. The research included measuring BMI, waist circumference, waist-to-height ratio (WHtR), screen time (i.e., duration spent on television, video games, and computer usage), and dietary habits (i.e., frequency of food consumption per week), as well as administering a validated questionnaire to evaluate the level of physical exercise. The correlations between obesity indices and lifestyle factors were analyzed by logistic regression. The logistic regression analysis indicated that being overweight or obese (based on BMI categories) or possessing abdominal obesity (according to WHtR categories) was significantly and inversely correlated with high levels of vigorous physical activity, regular consumption of breakfast and vegetables, and the restriction of sugary beverage intake (Al-Hazzaa et al., 2012).

Tharmin et al. (2021) utilized advanced ML approaches to predict obesity using publicly available health data from the Indonesian Basic Health Research. The aim of their research was to exceed traditional prediction models and identify an extensive array of risk factors for adult obesity utilizing easily accessible variables. The authors assessed the efficacy of machine learning techniques in identifying obesity by comparing three distinct methodologies: Logistic Regression, Naive Bayes, and Classification and Regression Trees (CART). Furthermore, they utilized the Synthetic Minority Oversampling Technique to rectify data imbalance. The results demonstrated that the Logistic Regression approach displayed the most efficacy, attaining an accuracy rate of roughly 72%. The authors indicated that various characteristics were significantly correlated with adult obesity, including marital status, geographical region, age category, and educational attainment. Consumption of sugary beverages, grilled foods, seasoning powders, fatty or oily foods, soft or carbonated drinks, alcoholic beverages, preserved foods, mental-emotional disorders, physical activity, smoking, diagnosed hypertension, and intake of fruits and vegetable (Thamrin et al., 2021).

Seyla et al. investigated the classification of obesity based on food and physical activity patterns via ML methods, concluding that the support vector machine (SVM) surpassed other classifiers in performance (Lundberg and Lee, 2017). Jindal et al. conducted ensemble ML methods for predicting obesity based on the primary determinants: age, height, weight, and BMI. The ensemble model employed Random Forest (RF), a generalized linear model, and partial least squares, achieving a prediction accuracy of 89.68% (Selya and Anshutz, 2018). Golino et al. employed a machine learning method, namely, a classification tree, to examine the prediction of elevated blood pressure based on BMI, waist circumference (WC), hip circumference (HC), and waist–hip ratio (WHR) in a cohort of 400 college students aged 16–63 years (56.3% female). The model surpassed the conventional logistic regression model for predicting efficacy. The model exhibited a sensitivity of 80.86% and specificity of 81.22% in the training set, and 45.65% and 65.15% in the test sample for women. The sensitivity was 72% and specificity was 86.25% in the training set, and 58.38% and 69.70% in the test set for men, respectively (Golino et al., 2014).

Prior research on obesity prediction using ML has often focused on either conventional classifiers or clinical datasets with limited behavioral diversity, with minimal integration of explainability mechanisms. Almeida et al. (2016) and Chen et al. (2015) used neural networks or SVMs for body fat or overweight classification but did not provide model interpretability beyond raw accuracy. While Gupta et al. (2022) applied LSTM models on longitudinal EHRs, they acknowledged the “black box” nature of deep learning. More recent studies such as those by Lin et al. (2023), Du et al. (2024), and Lian et al. (2025) have employed SHAP to enhance interpretability, but their work primarily emphasized either global feature importance or limited case-specific visualizations. Our study advances this body of work by not only employing a wide variety of classification models on lifestyle and behavioral data but also systematically integrating and comparing two complementary XAI methods—SHAP (SHapley Additive exPlanations) and LIME (Local Interpretable Model-agnostic Explanations) —for both local and global interpretability. This dual-method approach, combined with repeated holdout evaluations and diverse lifestyle predictors, offers a more transparent, rigorous, and generalizable framework for obesity risk assessment. Furthermore, our methodological contribution includes a comparative evaluation of fidelity, sparsity, and consistency—metrics often underexplored in previous literature.

This research tackles this deficiency by formulating and assessing an innovative ML-based method combined with XAI techniques for predicting obesity. Our study utilizes six different ML models: Bernoulli Naive Bayes, CatBoost, Decision Tree, Extra Trees Classifier, Histogram-based Gradient Boosting, and Support Vector Machine. Each model is associated with two notable XAI approaches - SHAP and LIME - to facilitate both local and global interpretability of the predictions. The main contributions of this study are summarized as follows:

1) The first contribution of this study is the development of various classification algorithms for predicting obesity levels and their subsequent explanation using two distinct XAI methods.
2) The second contribution lies in identifying the attributes that significantly influence the training of machine learning models for obesity level prediction, employing both local and global explanation techniques.
3) Finally, the third contribution involves evaluating and comparing the performance of the XAI methods in predicting obesity levels based on the proposed evaluation metrics.

This study seeks to enhance the field by offering a more thorough and interpretable methodology for predicting obesity. The results may enhance the development of more effective, tailored intervention options and deepen our comprehension of obesity risk factors. The methodology established in this study may function as a framework for deploying ML solutions in further healthcare applications where transparency and interpretability are essential.

## 2 Materials and methods

### 2.1 Participants

The present study employed an observational design and received approval from the Inonu University Health Sciences Non-Interventional Clinical Research Ethics Committee (approval number: 2024/5989). It utilized open access data to assess obesity levels among 498 participants aged between 14 and 61 years, considering their eating habits and physical activity patterns (Palechor and De la Hoz Manotas, 2019). The dataset comprised 17 features gathered from an online survey filled out anonymously. Eating-related variables included frequent high-calorie food consumption (FAVC), vegetable consumption frequency (FCVC), number of main meals per day (NCP), food intake between meals (CAEC), daily water intake (CH20), and alcohol use (CALC). Physical condition variables covered calorie tracking (SCC), frequency of physical activity (FAF), time spent on electronic devices (TUE), and type of transportation used (MTRANS). Additional variables such as gender, age, height, and weight were also collected. After calculating the BMI for each participant, obesity levels were classified based on WHO criteria: underweight (<18.5), normal (18.5–24.9), overweight (25.0–29.9), obesity I (30.0–34.9), obesity II (35.0–39.9), and obesity III (>40). Detailed information about the dataset properties is provided in the Supplementary Material.

### 2.2 Proposed methods

**FIGURE 1:**
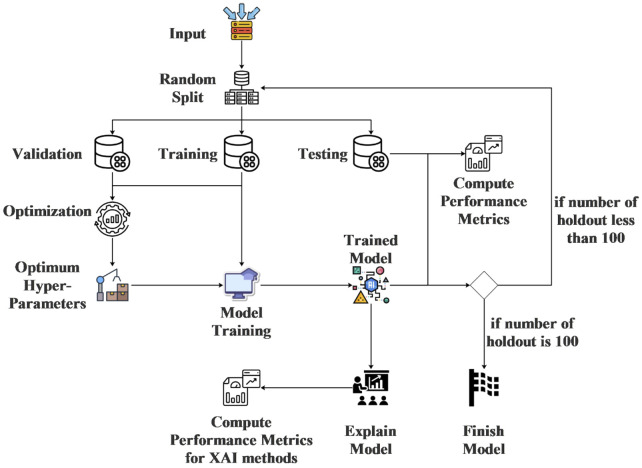
Flow diagram of analysis stages in this study.

In this study, several ML models, which are Bernoulli Naive Bayes, CatBoost, Decision Tree Classifier, Extra Trees Classifier, Histogram-Based Gradient Boosting Classifier and Support Vector Machine, are proposed to predict obesity levels based on physical activity and eating habit features. The selection of ML models in this study was motivated by their diverse algorithmic approaches, proven effectiveness in classification tasks, and their ability to handle both categorical and numerical features present in our dataset. Bernoulli Naive Bayes was chosen for its simplicity and efficiency with binary features, while CatBoost and Histogram-Based Gradient Boosting were included due to their robustness in handling categorical data and resistance to overfitting. Decision Trees and Extra Trees were selected for their interpretability and ensemble advantages, whereas Support Vector Machines were included for their capability to model complex boundaries via kernel functions. This variety ensures a comprehensive evaluation of model performance across different algorithmic paradigms. The hyper-parameters of the proposed models are optimized using a random search technique. To ensure the reliability of the results, repeated holdout tests are conducted. Additionally, to assess the importance of the features used by the models in making predictions, each model is explained using two distinct XAI methods. While the use of XAI methods has gained popularity, determining which method is superior remains a contentious issue. Therefore, the XAI models employed in this study were compared for each classification model using various evaluation metrics. For explainability, both SHAP and LIME were employed to address potential biases inherent in relying on a single XAI method. SHAP, grounded in game theory, provides global interpretability by quantifying feature importance consistently across the dataset, while LIME offers local, model-agnostic explanations for individual predictions. SHAP is generally more popular in research due to its mathematical rigor, but LIME’s simplicity makes it accessible for real-time applications. Performance-wise, SHAP tends to be computationally heavier but more consistent, whereas LIME may vary in fidelity depending on perturbation settings. By comparing both methods, we aim to mitigate methodological limitations and provide a balanced assessment of feature importance. The flow diagram of the analysis stages in this study is shown in Figure 1.

As illustrated in Figure 1, the experimental process began with the selection of random samples from the dataset, resulting in the creation of three distinct datasets: training, test, and validation sets. The random sampling process was conducted in a stratified manner to ensure that each subset (training, test, and validation) maintained a balanced representation of all obesity-level classes present in the original dataset. Specifically, the data was partitioned such that the proportion of classes in each subset mirrored the distribution of the full dataset. This approach mitigates potential biases introduced by random sampling and enhances the generalizability of the model evaluations. The sampling was performed without replacement to guarantee that no data point appeared in more than one subset, thereby maintaining independence between the splits. Subsequently, the hyper-parameters of the classification model were optimized using the training and validation datasets. For this purpose, random search was employed to optimize the hyper-parameters of each classification model individually. Following the optimization, the final model was trained using the optimum hyper-parameters, and its performance scores were evaluated on the test dataset. Additionally, model explanations were generated using XAI methods, and the performance scores of these methods were also calculated. This entire process was repeated 100 times, yielding 100 distinct performance scores. The repeated holdout method was chosen to mitigate potential biases arising from random data partitioning and to ensure robust evaluation of model performance. By repeating the holdout process 100 times, we account for variability in training-test splits, thereby reducing the risk of overfitting and providing a more reliable estimate of model generalizability. Additionally, class balance was maintained across all splits to prevent skewed performance metrics. This approach aligns with best practices in machine learning, particularly when dealing with moderate-sized datasets, as it enhances the stability of performance estimates without requiring computationally intensive cross-validation.

#### 2.2.1 Classification methods

##### 2.2.1.1 Bernoulli Naive Bayes

Bernoulli Naive Bayes (BNB) is a probabilistic classifier that works based on Bayes’ theorem with strong independence assumptions between the features. Each feature is modeled as a Bernoulli variable, assuming its presence or absence contributes independently to the likelihood of a class. Despite its simplicity, it performs well in high-dimensional data scenarios, especially when features are binary. However, it may struggle with complex, non-linear relationship (Murphy, 2012).

##### 2.2.1.2 CatBoost

CatBoost is an implementation of gradient boosting designed specifically to handle categorical data without requiring extensive preprocessing, such as one-hot encoding. The model efficiently incorporates categorical variables through its internal encoding mechanism. It also reduces the chances of overfitting by utilizing ordered boosting, which uses subsets of training data to build models iteratively, preventing bias from earlier predictions. CatBoost often outperforms other boosting algorithms on datasets with mixed data types due to its efficiency in managing categorical features and handling large-scale datasets. It has demonstrated strong performance in ranking, regression, and classification tasks (Prokhorenkova et al., 2018).

##### 2.2.1.3 Decision Tree Classifier

Decision Tree (DT) is a non-parametric supervised learning algorithm used for both classification and regression tasks. DT works by recursively partitioning the feature space into homogenous subsets based on feature values that maximize a chosen metric like Gini impurity or information gain. The tree is constructed from a root node, where each split represents a decision rule leading to terminal leaves that correspond to class labels. One of the main advantages of decision trees is their interpretability, as they provide an easy-to-understand representation of the decision-making process. However, they are prone to overfitting, especially when deep trees are constructed (Quinlan, 1986).

##### 2.2.1.4 Extra Trees Classifier

The Extra Trees Classifier (ETC) is an ensemble method similar to Random Forest, but it introduces more randomness in the tree construction process. Unlike traditional decision trees, where the best split is determined from the data, Extra Trees selects splits randomly. This additional randomness leads to increased variance reduction and generally better generalization performance, particularly when dealing with noisy data. It is computationally efficient as it reduces the variance without requiring bootstrap sampling. Extra Trees is particularly useful in high-dimensional datasets due to its resilience against overfitting compared to single decision trees (Geurts et al., 2006).

##### 2.2.1.5 Histogram-Based Gradient Boosting Classifier

The Histogram-Based Gradient Boosting Classifier (HistGB) is an efficient implementation of the gradient boosting algorithm that discretizes continuous features into integer-based histograms to reduce the memory footprint and computation time. This approach enhances performance, particularly for large datasets, by enabling faster training and inference compared to traditional gradient boosting methods. The model constructs weak learners, such as typically decision trees, in a stage-wise manner, focusing on correcting the errors made by previous models. HistGB is particularly useful for tabular data and has been shown to deliver robust results in both classification and regression tasks (Tamim Kashifi and Ahmad, 2022; Ke et al., 2017).

##### 2.2.1.6 Support vector machine

Support Vector Machine (SVM) is a supervised learning algorithm used for classification and regression tasks. SVM aims to find the optimal hyperplane that best separates the classes in the feature space by maximizing the margin between the nearest data points of different classes, called support vectors. SVM is particularly effective in high-dimensional spaces and is versatile with both linear and non-linear decision boundaries, thanks to the kernel trick, which allows it to work efficiently in a transformed feature space. It is robust to overfitting, particularly in high-dimensional datasets, though it can be computationally intensive on large datasets (Cortes and Vapnik, 1995).

#### 2.2.2 Explainable artificial intelligence

##### 2.2.2.1 Shapley additive explanations

SHapley Additive exPlanations (SHAP) is a game-theoretic approach used to explain the output of machine learning models by attributing each feature’s contribution to the final prediction. SHAP values are based on the Shapley value concept from cooperative game theory, which ensures fair allocation of the prediction’s contribution among all features. For any model prediction, SHAP values measure how each feature contributes to moving the prediction away from a baseline value. The main advantage of SHAP is its consistency and local accuracy, meaning that features contributing more to the prediction are assigned larger SHAP values. SHAP ensures that the sum of individual feature contributions equals the difference between the model’s output and the baseline. Additionally, SHAP values provide both global and local interpretability, allowing users to understand individual predictions and general feature importance across the dataset. While SHAP provides a unified measure of feature importance for any machine learning model, one downside is its computational complexity, especially for large datasets or highly complex models. SHAP is commonly used for black-box models like gradient boosting machines, random forests, and deep neural networks, making it a versatile tool for model interpretability (Lundberg and Lee, 2017).

##### 2.2.2.2 Local Interpretable Model-agnostic explanations

Local Interpretable Model-agnostic Explanations (LIME) is a model-agnostic technique designed to explain individual predictions of any black-box machine learning model by approximating the local decision boundary of the model with a simpler, interpretable model. It works by perturbing the input data instance and then observing how the black-box model’s predictions change. LIME then fits a local surrogate model, typically a linear model or decision tree, on this perturbed data to approximate the decision boundary around that specific instance. The simplicity of the surrogate model makes it interpretable and enables the user to understand the most influential features contributing to that particular prediction. One of LIME’s key strengths is that it can be applied to any machine learning model, making it extremely flexible. However, because it only focuses on local interpretability, it does not provide insights into the global behavior of the model. Another limitation of LIME is the randomness introduced by the perturbation process, which can lead to slightly different explanations across multiple runs unless the perturbation settings are carefully managed. Despite this, LIME is widely used in practice for understanding individual predictions of models like deep learning networks, ensemble methods, and other non-transparent algorithms (Ribeiro et al., 2016).

#### 2.2.3 Random search

Random search is a popular method for hyper-parameter optimization that selects values for hyper-parameters randomly from predefined distributions. Unlike grid search, where all possible combinations of hyper-parameters are exhaustively tested, random search samples a fixed number of random combinations from the hyper-parameter space. This allows the algorithm to explore a broader set of configurations more efficiently. One of the main advantages of random search is that it tends to find better models with fewer iterations, especially when only a small subset of hyper-parameters has a significant impact on model performance. In high-dimensional spaces, grid search can become computationally expensive, whereas random search can provide similar or better results with much less computation, particularly when certain hyper-parameters are more important than others. Random search is also easier to parallelize, as each trial is independent, and its flexibility allows practitioners to define different distributions for each hyper-parameter. Random search is particularly useful for complex models, such as deep learning networks and ensemble methods, where the number of hyper-parameters is large. However, random search does not guarantee finding the global optimum, as it does not systematically cover the search space. Despite this, it often performs well in practice, especially when coupled with techniques like cross-validation to ensure robust model evaluation (Bergstra and Bengio, 2012).

#### 2.2.4 Performance metrics

##### 2.2.4.1 Accuracy

Accuracy (acc) is a widely used evaluation metric for classification models and represents the proportion of correctly classified instances out of the total number of instances. Mathematically, it is defined as the sum of true positives and true negatives divided by the total number of predictions. While accuracy is simple and intuitive, it can be misleading in cases of imbalanced datasets. Accuracy is most effective when the dataset is balanced and when the cost of false positives and false negatives is comparable (Powers, 2020).

##### 2.2.4.2 Precision

Precision (pre), also known as positive predictive value, is a metric that quantifies the accuracy of positive predictions made by a model. It is defined as the ratio of true positives to the sum of true positives and false positives. Precision is particularly important in situations where false positives are costly or undesirable, such as in medical diagnoses, where incorrectly identifying a patient as having a disease may lead to unnecessary treatments. A high precision value indicates that the model makes few false positive errors, meaning that when it predicts a positive outcome, it is likely to be correct (Powers, 2020).

##### 2.2.4.3 F1 score

The F1 score (f1) is the harmonic mean of precision and recall, providing a single metric that balances both concerns. It is particularly useful when dealing with imbalanced datasets, where high precision may come at the cost of low recall or *vice versa*. By combining precision and recall, the f1 ensures that both false positives and false negatives are taken into account. A perfect f1 of 1.0 indicates that both precision and recall are maximized, whereas a lower f1 indicates a trade-off between these metrics. The f1 is most useful when the cost of false positives and false negatives are similar and when there is a need to balance precision and recall (Powers, 2020).

##### 2.2.4.4 Area under the ROC curve

The Area under the Receiver Operating Characteristic (ROC) Curve (AUC) measures the ability of a model to distinguish between classes and is based on the ROC curve, which plots the true positive rate against the false positive rate at various threshold levels. The AUC value ranges from 0 to 1, where a value of 1 represents a perfect model, 0.5 indicates random guessing, and values below 0.5 suggest a model performing worse than random. AUC is particularly useful when dealing with imbalanced datasets, as it is less affected by class distribution than metrics like accuracy. AUC provides an aggregate measure of performance across all classification thresholds, making it effective for comparing models. AUC is especially valuable in situations where the trade-off between sensitivity and specificity is critical, such as in medical diagnostics or fraud detection (Powers, 2020).

##### 2.2.4.5 Fidelity

Fidelity is a key metric used to assess how closely an explanation provided by an XAI method aligns with the behavior of the original model. Specifically, fidelity measures the extent to which the explanations reflect the true decision-making process of the underlying black-box model. High fidelity indicates that the explanations are faithful to the model’s predictions, meaning that the interpretable model used to generate explanations closely mimics the behavior of the original complex model. Fidelity is crucial because an explanation is only useful if it accurately represents the model’s reasoning process. In this study, fidelity is calculated based on the compatibility between the globally obtained feature ranking and the locally calculated features. The fidelity score ranges from 0 to 1, with values closer to 1 indicating higher success of the method (Ribeiro et al., 2016).

##### 2.2.4.6 Sparsity

Sparsity refers to the degree to which an explanation is concise and focuses on a minimal set of important features. A sparse explanation is easier to understand and interpret because it emphasizes only the most relevant factors contributing to the model’s decision. Sparsity is particularly important for models with high-dimensional input spaces, where explanations could otherwise become overwhelming or cluttered with irrelevant details. By limiting the number of features included in the explanation, sparsity enhances interpretability without sacrificing too much information. XAI methods like LIME or SHAP can be tuned to produce sparser explanations by adjusting their regularization or threshold settings, focusing on fewer influential features. In this study, the average attribute importance score was used as the threshold for calculating sparsity. Sparsity represents the number of attributes with scores higher than this average (Rudin, 2019).

##### 2.2.4.7 Consistency

Consistency is a metric that evaluates whether an XAI method provides stable and reliable explanations across different instances or models. In other words, an XAI method is consistent if the explanations remain coherent when the underlying model or the data slightly changes. Consistency ensures that small variations in input data or model parameters do not lead to drastically different explanations, which is important for building trust in the model’s interpretability. Consistent explanations also help ensure that users can rely on the model across various scenarios without constantly questioning its rationale. Lack of consistency can lead to confusion and reduce trust in the model’s predictions, especially in high-stakes domains such as healthcare or criminal justice. In this study, consistency was calculated by measuring the L1 distance between the global explanations generated for each holdout set (Lundberg and Lee, 2017).

## 3 Experiment results

In the experimental phase of this study, six different classification models for obesity level prediction were analyzed, with each model explained using two different XAI methods. To ensure the significance of the results, a repeated holdout approach was employed. In the first step, the dataset was divided into three subsets: training, testing, and validation sets. Specifically, 20% and 10% of the samples were randomly selected to form the testing and validation datasets, respectively, while the remaining samples were used to generate training dataset. To facilitate repeated holdout, this process was repeated 100 times, resulting in 100 distinct training, testing, and validation datasets. The training sets were used to train the models, the validation sets were used to evaluate model performance during hyper-parameter optimization, and the testing sets were used to assess the final model’s performance. Hyper-parameter optimization and model training were performed separately for each holdout. For hyper-parameter optimization, the random search method was employed with the number of iterations set to 50. The optimized hyper-parameters, their types, and the search spaces for each model are detailed in Table 1.

**TABLE 1:** Details of the name, type, and space of the optimized hyper-parameter for each model.

Model name	Hyper-parameter name	Hyper-parameter type	Hyper-parameter space
BNB	alpha	Float	Low = 0, High = 1
	force_alpha	Boolean	[True, False]
	binarize	Float	Low = 0, High = 1
CatBoost	depth	Integer	Low = 3, High = 7
	iterations	Integer	Low = 50, High = 1000
	learning_rate	Float	Low = 0.001, High = 0.3
DT	criterion	Categorical	[“gini”, “entropy”, “log_loss”]
	max_depth	Integer	Low = 1, High = 10
	splitter	Categorical	[“best”, “random”]
ETC	criterion	Categorical	[“gini”, “entropy”, “log_loss”]
	max_depth	Integer	Low = 1, High = 10
	n_estimators	Integer	Low = 50, High = 1000
HGB	max_iter	Integer	Low = 3, High = 7
	max_leaf_nodes	Integer	Low = 5, High = 60
	learning_rate	Float	Low = 0.001, High = 1
	tol	Float	Low = 0.000001, High = 0.003
SVM	max_iter	Integer	Low = 10, High = 1000
	tol	Float	Low = 0.00001, High = 0.003
	C	Float	Low = 0.0001, High = 10

Table 2 presents the average values across holdouts for acc, pre, f1, and AUC. While high metric values indicate strong model performance, it is equally important for models to be robust. Consistency of results across holdouts is considered an indicator of robustness. In this regard, the standard deviation (std) values across holdouts for each model are also included in Table 2.

**TABLE 2:** Average performance scores of models that trained using optimal hyper-parameters.

Model name	acc	std acc	Pre	std pre	f1	std f1	AUC	std AUC
BNB	80.15%	0.49	75.14%	4.65	79.01%	0.87	82.97%	6.11
CatBoost	93.67%	1.37	92.36%	1.78	93.57%	1.49	99.39%	1.73
DT	91.64%	1.96	90.51%	2.08	91.00%	2.07	97.87%	2.82
ETC	85.75%	0.99	84.96%	3.60	85.90%	1.84	91.89%	4.79
HGB	89.58%	1.44	90.65%	2.52	90.22%	1.74	95.84%	1.32
SVM	81.49%	1.23	81.66%	3.30	84.86%	1.86	90.22%	1.70

Analysis of the results in Table 2 reveals that the CatBoost model achieved the highest performance across all metrics, with an accuracy of 93.67% ± 1.37%, precision of 92.36% ± 1.78%, F1-score of 93.57% ± 1.49%, and AUC of 99.39% ± 1.73%. In contrast, the BNB model exhibited the lowest performance, with an accuracy of 80.15% ± 0.49%, precision of 75.14% ± 4.65%, F1-score of 79.01% ± 0.87%, and AUC of 82.97% ± 6.11%. The SVM model also showed comparatively lower performance, particularly with an accuracy of 81.49% ± 1.23%. Regarding std values, the AUC metric exhibited higher variability across the models compared to other metrics. Although CatBoost achieved the best results particularly in the AUC metric, it maintained relatively low standard deviation values, indicating that CatBoost is both highly performant and robust.

**FIGURE 2:**
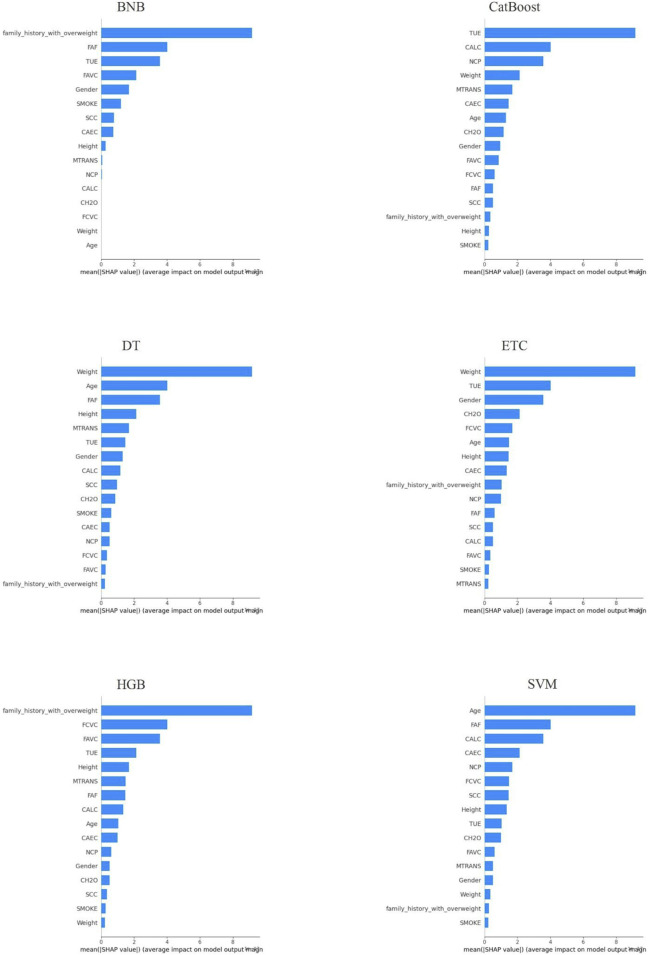
Local Explanation of Each Method’s Best Holdout Model using SHAP.

**FIGURE 3:**
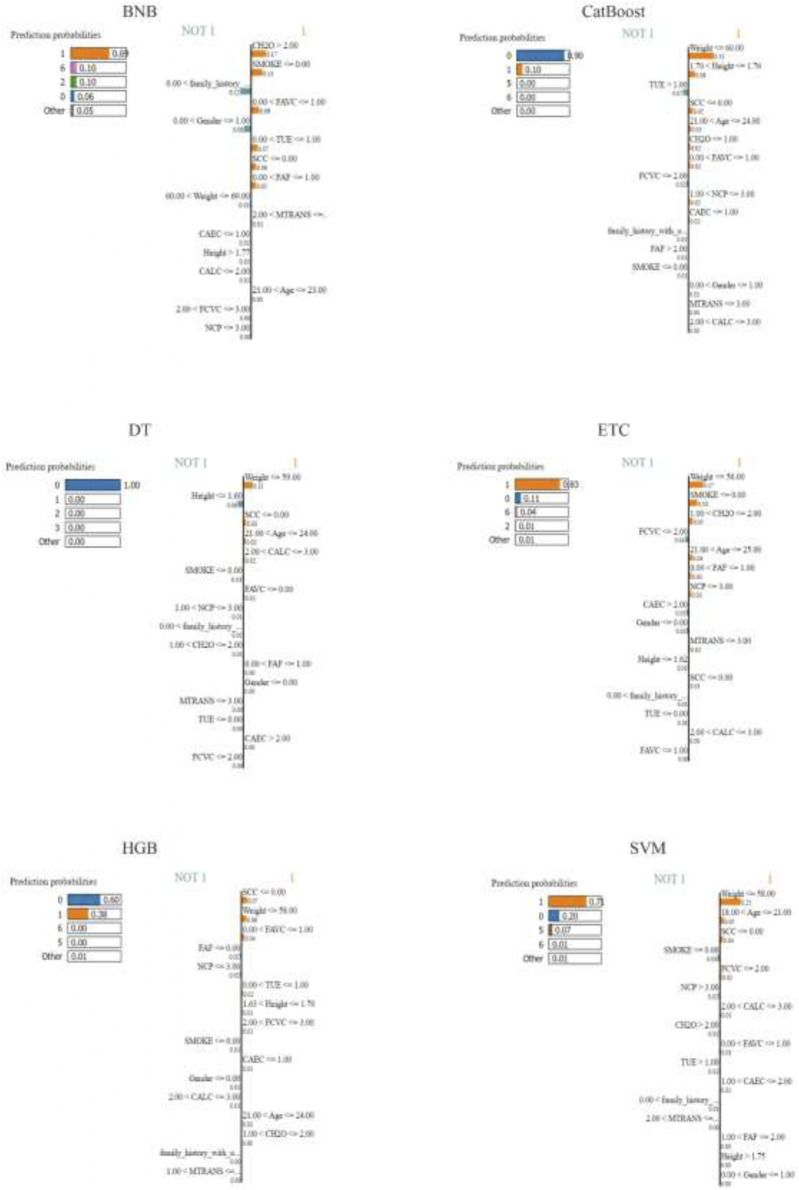
Local Explanation of Each Method’s Best Holdout Model using LIME.

Local plots were generated for a random sample of the test data using the highest scoring hold for the LIME annotations for each classification method. The annotation plots for the models are presented in Figures 2, 3 for SHAP and LIME, respectively.

Upon analyzing the local explanation graphs, it is evident that each model produces different explanation results, and the explanations for the same model vary across different XAI methods. While local explanations provide valuable insights, global explanations offer a more comprehensive understanding of attribute importance. Therefore, the attribute importance scores calculated across the holdouts were averaged to derive a final ranking of attributes. Table 3 presents the attribute importance rankings for each model, as determined by the LIME and SHAP methods, based on the average scores from the holdouts. After generating global explanations for the models, the SHAP and LIME methods calculated individual scores for each attribute. These scores indicate the relative importance of each attribute. In line with the proposed approach, 100 randomized datasets were created, and model explanations were generated for each. At this stage, the scores calculated for each attribute in every holdout iteration were summed and divided by 100 to compute the average attribute importance scores. Table 3 presents the ranking of attributes based on these computed importance scores.

**TABLE 3:** Feature importance rankings were determined based on the average global LIME and SHAP scores calculated across all holdout iteration.

XAI method	Classifier model name	Feature importance
SHAP	BNB	[“TUE”, “family_history_with_overweight”, “Gender”, “FAF”, “FAVC”, “MTRANS”, “SCC”, “CAEC”, “SMOKE”, “Weight”, “Height”, “CALC”, “Age”, “CH2O”, “FCVC”, “NCP”]
	CatBoost	[“Weight”, “Height”, “Age”, “FAF”, “TUE”, “CH2O”, “CAEC”, “CALC”, “FCVC”, “Gender”, “family_history_with_overweight”, “MTRANS”, “NCP”, “FAVC”, “SCC”, “SMOKE”]
	DT	[“Weight”, “Height”, “FAF”, “Age”, “CAEC”, “TUE”, “FCVC”, “family_history_with_overweight”, “Gender”, “FAVC”, “MTRANS”, “CH2O”, “CALC”, “NCP”, “SMOKE”, “SCC”]
	ETC	[“Weight”, “Height”, “Age”, “FAF”, “TUE”, “CH2O”, “family_history_with_overweight”, “CALC”, “Gender”, “FCVC”, “CAEC”, “MTRANS”, “FAVC”, “NCP”, “SCC”, “SMOKE”]
	HGB	[“Weight”, “Height”, “Age”, “FAF”, “TUE”, “CAEC”, “CH2O”, “family_history_with_overweight”, “FCVC”, “FAVC”, “CALC”, “Gender”, “NCP”, “MTRANS”, “SCC”, “SMOKE”]
	SVM	[“Weight”, “Height”, “Age”, “FAF”, “TUE”, “CH2O”, “CAEC”, “CALC”, “FCVC”, “Gender”, “family_history_with_overweight”, “MTRANS”, “NCP”, “FAVC”, “SCC”, “SMOKE”]
LIME	BNB	[“SMOKE”, “SCC”, “CH2O”, “FAVC”, “family_history_with_overweight”, “TUE”, “Gender”, “FAF”, “MTRANS”, “NCP”, “CAEC”, “Height”, “Age”, “Weight”, “CALC”, “FCVC”]
	CatBoost	[“SCC”, “Weight”, “NCP”, “MTRANS”, “SMOKE”, “FAVC”, “Height”, “CH2O”, “CAEC”, “family_history_with_overweight”, “TUE”, “CALC”, “FCVC”, “Age”, “Gender”, “FAF”]
	DT	[“Weight”, “SMOKE”, “Height”, “MTRANS”, “NCP”, “FAVC”, “FAF”, “SCC”, “FCVC”, “Age”, “CAEC”, “Gender”, “family_history_with_overweight”, “TUE”, “CH2O”, “CALC”]
	ETC	[“SMOKE”, “SCC”, “FAVC”, “Weight”, “NCP”, “MTRANS”, “family_history_with_overweight”, “CH2O”, “FCVC”, “CAEC”, “Age”, “TUE”, “FAF”, “CALC”, “Gender”, “Height”]
	HGB	[“Weight”, “SCC”, “MTRANS”, “NCP”, “FAVC”, “Height”, “CH2O”, “SMOKE”, “CAEC”, “family_history_with_overweight”, “Age”, “FCVC”, “TUE”, “CALC”, “Gender”, “FAF”]
	SVM	[“Weight”, “SCC”, “NCP”, “MTRANS”, “Age”, “SMOKE”, “FAVC”, “FCVC”, “CH2O”, “FAF”, “TUE”, “CALC”, “CAEC”, “Gender”, “family_history_with_overweight”, “Height”]

The results presented in Table 3 clearly show that the feature importance rankings differ between the LIME and SHAP methods. For instance, according to SHAP, “Weight” and “Height” are consistently among the top important features across models, whereas LIME results emphasize features like “SMOKE” and “SCC”. Comparing these XAI methods is critical to understanding which features are more meaningful for predicting obesity levels. To further investigate the quality of these explanations, the performance metrics of LIME and SHAP are detailed in Table 4.

**TABLE 4:** Performance metrics of XAI models.

Classifier model name	XAI method	Fidelity	Std of fidelity	Sparsity	Std of sparsity	Consistency	Std of consistency
BNB	SHAP	0.5605	0.0339	0.4237	0.0037	3.8484	0.8559
	LIME	0.6228	0.0260	0.2693	0.0556	4.3977	0.9435
CatBoost	SHAP	0.6100	0.0150	0.5100	0.0808	3.4103	0.8108
	LIME	0.6441	0.0211	0.2781	0.0561	5.3813	0.7808
DT	SHAP	0.5264	0.0603	0.2675	0.0696	4.5138	0.7064
	LIME	0.6203	0.0154	0.2206	0.0903	5.1212	0.8095
ETC	SHAP	0.6104	0.0150	0.5175	0.0739	4.6338	0.8607
	LIME	0.6440	0.0325	0.2725	0.0676	4.9179	1.0141
HGB	SHAP	0.5980	0.0217	0.4887	0.0698	3.7916	0.8425
	LIME	0.6396	0.0228	0.2681	0.0697	5.2121	0.8388
SVM	SHAP	0.6098	0.0159	0.5056	0.0755	4.0037	0.8165
	LIME	0.6353	0.0194	0.2662	0.0832	5.4608	0.9603

Among the evaluated metrics, fidelity and sparsity are expected to be closer to 1, while consistency is ideally close to 0. Analyzing the values in Table 4:

• For the BNB model, LIME achieved a fidelity of 0.6228 ± 0.0260, while SHAP had a fidelity of 0.5605 ± 0.0339. Regarding sparsity, LIME scored 0.2693 ± 0.0556 compared to SHAP’s 0.4237 ± 0.0037. The consistency values were 4.3977 ± 0.9435 for LIME and 3.8484 ± 0.8559 for SHAP.
• For the CatBoost model, LIME obtained a fidelity of 0.6441 ± 0.0211, sparsity of 0.2781 ± 0.0561, and consistency of 5.3813 ± 0.7808, while SHAP recorded a fidelity of 0.6100 ± 0.0150, sparsity of 0.5100 ± 0.0808, and consistency of 3.4103 ± 0.8108.
• For the DT model, LIME fidelity was 0.6203 ± 0.0154, sparsity 0.2206 ± 0.0903, and consistency 5.1212 ± 0.8095, whereas SHAP fidelity was 0.5264 ± 0.0603, sparsity 0.2675 ± 0.0696, and consistency 4.5138 ± 0.7064.
• For the ETC model, LIME had a fidelity of 0.6440 ± 0.0325, sparsity of 0.2725 ± 0.0676, and consistency of 4.9179 ± 1.0141. In contrast, SHAP fidelity was 0.6104 ± 0.0150, sparsity 0.5175 ± 0.0739, and consistency 4.6338 ± 0.8607.
• For the HGB model, LIME achieved a fidelity of 0.6396 ± 0.0228, sparsity of 0.2681 ± 0.0697, and consistency of 5.2121 ± 0.8388. SHAP achieved a fidelity of 0.5980 ± 0.0217, sparsity of 0.4887 ± 0.0698, and consistency of 3.7916 ± 0.8425.
• For the SVM model, LIME fidelity was 0.6353 ± 0.0194, sparsity was 0.2662 ± 0.0832, and consistency was 5.4608 ± 0.9603. SHAP, on the other hand, achieved a fidelity of 0.6098 ± 0.0159, sparsity of 0.5056 ± 0.0755, and consistency of 4.0037 ± 0.8165.

Overall, LIME consistently demonstrated higher fidelity scores compared to SHAP across all models, while SHAP generally showed better sparsity and consistency metrics. Additionally, the low standard deviations across all models and metrics indicate that the results are stable and robust.

## 4 Discussion

Although there are classical ML and XAI based studies on obesity level prediction in the literature, there is no research that integrates LIME at local level and SHAP at global level and evaluates each explanation method by further comparing using interpretability criteria such as fidelity, sparsity and consistency. While previous studies have examined ML models combined with XAI techniques for obesity prediction, our study advances the field in several important ways. Although several studies have applied ML and XAI to obesity-related data, our study distinguishes itself with a more comprehensive model comparison (six different ML classifiers), double-layer interpretability analysis.

Six different classification models were trained to predict obesity levels using the repeated retention method. The hyperparameters of the models were optimized using the random search method. Then, each model was explained both locally and globally using the LIME and SHAP methods. Experimental results show that the CatBoost model outperforms other models in predicting obesity levels. In terms of performance, the CatBoost model was followed by DT, HGB, ETC, SVM and BNB models. When the model explanations were analyzed, it was seen that the feature importance rankings differed among the XAI methods. According to the global explanation scores, the features that were consistently ranked in the top 10 for the first three models (CatBoost, DT and HGB) were “Age”, “Height”, “Weight”, “FCVC”, “CAEC”, “FAF” and “TUE” by SHAP and “Height”, “Weight”, “FAVC”, “NCP”, “SMOKE”, “SCC” and “MTRANS” by LIME. The features that were consistently ranked in the top 10 for all three models by both XAI methods were “Height” and “Weight”. When comparing the XAI methods, LIME consistently outperformed SHAP in the fidelity metric, while SHAP gave better results in the sparsity and consistency metrics. Additionally, both XAI methods demonstrated robustness by achieving relatively low standard deviation values ​​in all three metrics.

CatBoost has demonstrated high performance on complex datasets containing a mixture of categorical and continuous features, possible class imbalance, and nonlinear interactions between variables (Prokhorenkova et al., 2018; Akbulut et al., 2023). The performance ranking of the models is in line with other research showing the effectiveness of boosting techniques on similar health prediction tasks (Zhang et al., 2020; Guldogan et al., 2023; Yagin et al., 2023). The differences between the XAI methods are consistent with Lundberg and Lee’s (2017) observation that the results of SHAP and LIME may vary due to different annotation approaches (Lundberg and Lee, 2017).

A recent study by Khater et al. (2024), also used explainable AI (specifically SHAP) to investigate its impact on obesity classification. The authors focused only on a single machine learning model (Random Forest) and lacked a comparative analysis based on the performance of different classifiers or interpretability methods. Additionally, unlike Khater et al. (2024), who focused on SHAP, where Random Forest was primarily applied, our approach integrates both SHAP and LIME across multiple models and provides a more robust and generalizable framework by comparing XAI approaches with fidelity, sparsity, and consistency metrics. Azad et al. (2025) used a stacked ensemble model that included LGBM, Logistic Regression, and Random Forest with LIME as the sole interpretability method. Although their model achieved high accuracy (98.82%), their reliance on a single XAI method limited the depth and robustness of interpretability. The authors obtained local, i.e., patient-based, explanations of the model using LIME alone. Therefore, our approach provides a more holistic, transparent, and generalizable framework for understanding obesity risk through interpretable ML.

Lee et al. (2022) did a study that looked in depth at the genetic, epigenetic, and environmental factors that affect BMI and fat. The generalized multifactor dimensionality reduction method was used to do a genome-wide and epigenome-wide scan and look for links between a lot of SNPs, food and lifestyle factors, and DNA methylation sites. After finding statistically significant markers like genetic variations, epigenetic changes, and nutritional variables, the scientists used machine learning techniques to guess how obese people were in a separate test set. The authors looked closely at the genetic, epigenetic, and environmental factors that affect BMI and obesity in their study. They used the generalized multifactor dimensionality reduction method to do a genome-wide and epigenome-wide scan and look at how a lot of SNPs, DNA methylation sites, and food and lifestyle factors are connected. After finding statistically significant markers like genetic variations, epigenetic changes, and nutritional variables, the scientists used ML techniques to guess how obese people were in a separate test set.

When our XAI results are analyzed, LIME’s better performance in the fidelity metric confirms Ribeiro et al.'s (2016) claim that LIME is strong in terms of local accuracy (Ribeiro et al., 2016), while SHAP’s superiority in the consistency and sparsity metrics is in line with studies showing the effectiveness of this method in global annotation (Molnar, 2020). The fact that both XAI methods show low standard deviation values in all metrics is in line with previous studies emphasizing the reliability of these techniques (Adadi and Berrada, 2018; Yagin et al., 2024).

A meta-analysis study in the literature examined the performance of logistic regression (LR) and ML methods to predict obesity risk. The results of the study show that both LR and ML methods achieve equally good performance in predicting obesity, and they reported that there was no significant evidence that ML performed better than LR (Boakye et al., 2025).

Kalhori et al. reported that artificial intelligence techniques have the potential to predict obesity and reduce its complications. The authors reported in their study that supervised learning methodologies such as random forest and linear regression models are generally used to predict obesity in the literature. In these studies, demographic data is usually used for obesity prediction. Kalhori et al. reported in their published review that the ANN model achieved superior performance in terms of AUC and the k-means approach achieved superior performance in terms of accuracy within the scope of obesity prediction (Kalhori et al., 2025).

A review of the broader literature indicates that various ML models—ranging from traditional decision trees (Dugan et al., 2015), ensemble models (Lin et al., 2023; Shahabi et al., 2024), to advanced boosting methods (Du et al., 2024; Lian et al., 2025), have been effectively employed for obesity prediction using diverse data types including anthropometrics, EHRs, and biochemical markers. Lin et al. utilized SHAP to explain factors like waist circumference (WC) and systolic blood pressure (SBP), while Lin et al. (2023) highlighted individual-level explanations on biochemical data, pointing to the need for personalized insights. Gupta et al. (2022) applied deep learning on large-scale pediatric data, yet recognized its limited interpretability. In contrast, our study offers a more holistic evaluation framework combining behavioral and lifestyle predictors with interpretable ML outputs. This aligns with a growing consensus in the literature advocating for explainable models to bridge the clinical adoption gap. By synthesizing insights from both global and local explanation perspectives, and reporting multiple interpretability metrics, our approach complements and extends previous findings in a methodologically rigorous and practically meaningful direction.

The important variables determined by SHAP and LIME methods largely overlap with the risk factors frequently emphasized in the obesity literature. In particular, variables such as “weight”, “height”, “age”, “physical activity frequency (FAF)”, “screen time (TUE)” and “caloric intake behavior (CAEC)”, which stand out according to SHAP analysis, can be evaluated within the scope of physiological risk factors. Anthropometric measurements such as weight, height and age are the main physiological determinants in predicting obesity by directly affecting body composition. In addition, low physical activity frequency and high screen time negatively affect energy balance and increase the risk of obesity. These findings are consistent with lifestyle-based physiological factors, especially emphasized in studies such as Al-Hazzaa et al. (2012), and Thamrin et al. (2021). On the other hand, variables such as “SMOKE”, “SCC” (calorie tracking), “family_history_with_overweight”, “number of meals (NCP)” and “type of transportation (MTRANS)”, which are found important by LIME, can be evaluated in a sociocultural context. Smoking and calorie tracking habits are related to the individual’s level of health awareness; this situation is shaped by factors such as social environment, education level and health literacy (Khater et al., 2024; Thamrin et al., 2021).

In addition, the presence of overweight individuals in the family is a sociocultural factor in terms of both genetic predisposition and the transmission of similar lifestyles. Transportation preference (walking, private vehicle, etc.) is an environmental variable affecting the individual’s physical activity level and is a reflection of urbanization and lifestyle patterns (Gupta et al., 2022). In general, the variables used in the study are meaningful explanatory factors at both physiological and sociocultural levels and are compatible with contemporary approaches that address obesity in a multidimensional manner in these aspects. The results obtained are consistent with the risk factors reported by the World Health Organization (WHO) and similar authorities and support the applicability of predictive models in the field (Lim et al., 2020; Afolabi et al., 2023).

In conclusion, this work provides a thorough methodology for estimating obesity levels through the utilization of integrated ML models combined with XAI techniques. The study demonstrates the CatBoost model’s greater efficacy in assessing obesity levels, achieving 93.67% of the previously documented results in the literature. This conclusion indicates that algorithms are effective in analyzing the intricate relationships between CatBoost, lifestyle factors, and obesity outcomes. The comparative research of these approaches indicates that it demonstrates greater faithfulness in LIME, while the SHAP exhibits enhanced consistency. This synergistic performance indicates that employing both strategies concurrently can yield more thorough and dependable answers. The identification of fundamental predictive attributes via XAI approaches underscores the essential significance of both physical characteristics and lifestyle habits in the assessment of obesity. This discovery aligns with current medical knowledge and offers quantitative reinforcement for established obesity prevention techniques. Minimal standard deviation values across many assessment measures indicate the trustworthiness and stability of both calculated models and descriptions, suggesting their potential use in clinical settings. This research demonstrates that the integration of powerful ML techniques with XAI methodologies can produce robust and interpretable tools for obesity estimation. The findings advance the technical development of predictive health analytics and enhance the practical comprehension of obesity risk variables, hence facilitating possibly more effective and personalized intervention techniques.

While this study provides valuable insights into obesity prediction using ML and XAI, certain limitations must be acknowledged. The cross-sectional structure of the data precludes the monitoring of temporal changes in obesity levels. The sample size of approximately 500 individuals, though sufficient for initial model development, may restrict the generalizability of findings to broader populations. Larger and more diverse datasets would help validate the robustness of the identified predictors. Additionally, the inclusion of only 17 features, while informative, may not capture the full complexity of obesity determinants. Future research could benefit from incorporating additional variables such as genetic predispositions, metabolic markers, and environmental factors to enhance predictive accuracy and clinical relevance.

## Data Availability

The original contributions presented in the study are included in the article/Supplementary Material, further inquiries can be directed to the corresponding author.
