# Supplementary material for: Prediction of obesity levels based on physical activity and eating habits with a machine learning model integrated with explainable artificial intelligence
Source: Front Physiol. 2025 Jul 16;16:1549306. doi: 10.3389/fphys.2025.1549306 (PMC12308079; doi:10.3389/fphys.2025.1549306)
Supplement: Supplementary file 1 [file Supplementaryfile1.docx]

Dataset Characteristics

The dataset in this study has 17 variables, explained below:

Gender: categorical variable that shows the biological sex of the individual (male or female).

Age: numerical variable that shows the individual’s age in years.

Height: numerical variable that shows the individuals’ height in meters.

Weight: numerical variable that shows the individuals’ weight in kilograms.

Family history of overweight: categorical variable that shows if the individual has a family member who is overweight or obese (yes or no).

Frequently consumed high-calorie food (FAVC): categorical variable that shows if the individual often eats high-calorie food (yes or no).

Frequency of consumption of vegetables (FCVC): ordinal variable that shows how often the individual eats vegetables (1 = never, 2 = sometimes, 3 = always).

Number of main meals (NCP): ordinal variable that shows how many main meals the individual has daily (1 = between 1 and 2, 2 = three, 3 = more than three, 4 = no answer).

Consumption of food between meals (CAEC): ordinal variable that shows how often the individual eats food between meals (1 = no, 2 = sometimes, 3 = frequently, 4 = always).

SMOKE: categorical variable that shows whether the individual smokes or not (yes or no).

Consumption of water daily (CH2O): ordinal variable that shows how much water the individual drinks daily (1 = less than a liter, 2 = between 1 and 2 L, 3 = more than 2 L).

Monitor calorie intake (SCC): categorical variable that shows if the individual keeps track of their caloric intake (yes or no).

Frequency of physical activity (FAF): ordinal variable that shows how often the individual does physical activity (1 = never, 2 = once or twice a week, 3 = two or three times a week, 4 = four or five times a week).

Time using electronic devices (TUE): ordinal variable that shows how long the individual uses electronic devices (0 = none, 1 = less than an hour, 2 = between one and three hours, 3 = more than three hours).

Consumption of alcohol (CALC): ordinal variable that shows how often the individual drinks alcohol (1 = no, 2 = sometimes, 3 = frequently, 4 = always).

Type of transportation used (MTRANS): categorical variable that shows what kind of transportation the individual uses (automobile, motorbike, bike, public transportation, walking).

Level of obesity according to body mass index (NObesity): ordinal variable that shows the obesity level of the individual according to their BMI (insufficient weight normal weight, overweight level I, overweight level II, obesity type I, obesity type II, obesity type III).

After performing all the calculations to compute the BMI of each participant, the WHO criteria were applied to classify the obesity levels as follows: underweight = BMI less than 18.5; normal = BMI between 18.5 and 24.9; overweight = BMI between 25.0 and 29.9; obesity I = BMI between 30.0 and 34.9; obesity II = BMI between 35.0 and 39.9; and obesity III = BMI higher than 40. The WHO criteria are based on the relationship between BMI and the risk of chronic diseases and mortality.

Table 1 reports the distribution of obesity levels.

Table 1. Distribution of obesity levels in the original dataset

| **Obesity Levels** | ***n*** | **%** |
| --- | --- | --- |
| Underweight | 34 | 6.80 |
| Normal Weight | 287 | 57.60 |
| Overweight Level I | 47 | 9.40 |
| Overweight Level II | 11 | 2.20 |
| Obesity Type I | 3 | 0.60 |
| Obesity Type II | 58 | 11.60 |
| Obesity Type III | 58 | 11.60 |
| Total | 498 | 100.00 |
